# Role of a multidisciplinary team in administering radiotherapy for esophageal cancer

**DOI:** 10.1186/s12885-020-07467-z

**Published:** 2020-10-08

**Authors:** Shengguang Zhao, Weixiang Qi, Jiayi Chen

**Affiliations:** Department of Radiation Oncology, Ruijin Hospital, Shanghai Jiao tong University School of Medicine, 197 Second Ruijin Road, Shanghai, 200025 China

**Keywords:** Esophageal neoplasms, Multidisciplinary team, Radiotherapy, Chemotherapy, Survival

## Abstract

**Background:**

Radiotherapy (RT) is the major part of the treatment strategy set by a multidisciplinary team (MDT) for patients diagnosed with esophageal cancer (EC). The effect of an MDT collaboration on patients with EC who underwent RT is unclear.

**Methods:**

We retrospectively collected all patients diagnosed with EC in the radiation oncology department at our institution from January 2015 to May 2017. The patients were divided into groups based on if they had their cases presented or not presented at the MDT meeting (with MDT and non-MDT, respectively). Propensity score matching (PSM) was applied at a ratio of 1:1 and the nearest neighbor matching method to compare the two groups.

**Results:**

A total of 212 consecutive patients were analyzed, including 157 with MDT and 55 non-MDT. In the unmatched population, the patients with MDT were more likely to received chemotherapy than the non-MDT patients (84.7% vs. 69.1%; × 2 = 6.373; *P* = 0.012).MDT-patients had significantly improved overall survival compared with non-MDT patients (*p* = 0.025). In the multivariate analysis, MDT was an independent prognostic factor for OS in patients with EC who underwent RT (*P* = 0.019, HR 0.59, 95% CI 0.38–0.92). After PSM for baseline characteristics, the benefit of MDT for OS became more obvious. Additionally, we also found that MDT was an independent predictor of receiving chemotherapy by using logistic regression analysis.

**Conclusion:**

In patients who underwent radiotherapy for esophageal cancer, MDT was an independent factor for overall survival, which probably due to the selection of multimodality treatment when compared to non-MDT setting.

## Background

EC is still one of the most lethal malignancies worldwide with a 5-year survival rate ranging from 15 to 25% [1]. Although the treatment of EC remains a challenge, the guidelines encourage the application of combined modality therapy for EC patients to achieve optimal treatment [2]. The MDT may include thoracic surgeons without limitations, medical oncologists, radiation oncologists, gastroenterologists, radiologists, and pathologists. Disease management by an MDT improved the staging accuracy, treatment selection and outcomes after surgery for EC patients [3, 4]. However, for EC patients who underwent RT, the role of an MDT remains unclear.

## Methods

### Study population

The multidisciplinary team (MDT) for esophageal cancer at Ruijin Hospital was established in 2013 with the aim of working together to generate a comprehensive treatment regimen for our esophageal cancer patients to achieve the best survival outcome. The MDT constituted a multi-disciplinary specialists comprising of thoracic surgeons, radiation oncologists, medical oncologists, radiologists, pathologists, gastroenterologist and specialist nurses. We retrospectively collected consecutive patients with EC who underwent RT in the radiotherapy department of our institution between January 2015 and May 2017. Depending on the referral source, the patients were divided into groups based on if they had their cases discussed or not discussed in an MDT meeting (with MDT and non-MDT, respectively).

In China, squamous cell carcinoma (SCC) is the predominant histologic subtype [5, 6], so we allocated adenocarcinoma and other histologic subtypes into one group. Patients who underwent transthoracic esophagectomy and lymphadenectomy with curative intent were grouped as patients with surgery, while those who underwent gastrostomy or jejunostomy with palliative intent and those who did not undergo surgery were grouped as non-surgery. Chemotherapy regimens based on cisplatin/5-FU or taxanes/platinum were decided upon by a radiotherapy oncologist or medical oncologist. Due to the characteristics of retrospective studies, neoadjuvant therapy had not yet been routinely carried out at our center for EC patients at the time of enrollment. Adjuvant radiotherapy is recommended for patients whose tumor stage was higher than T3 and whose lymph node or margin status was positive by pathological confirmation. All patients received radiotherapy at a dose of 50.4 Gy–54 Gy for adjuvant treatment, 30–40 Gy for palliative treatment, and 50.4–64.8 Gy for curative treatment. Patients with EC were staged in accordance with the American Joint Committee on Cancer TNM classification of malignant tumors, seventh edition. The pathological stage for patients who underwent curative operations and the clinical stage for the nonsurgery patients were recorded.

### Statistical analysis

OS was defined as the time from the date of surgery to the date of death for patients who underwent surgery or from the date of histologic or cytologic diagnosis to the date of death for patients who did not undergo surgery; patients who were alive on the date of last follow-up were censored.

Statistical analyses included chi-square tests for categorical variables, t-tests to compare quantitative variables, the Kaplan-Meier method to construct survival curves, the log-rank tests to compare survival curves, logistic regression modeling for odds ratios (ORs), and Cox’s proportional hazards model for multivariate analyses of the prognostic factors. A propensity score matching (PSM) analysis with 1:1 matching and the nearest neighbor matching method with a caliper of 0.2 was conducted to ensure well-balanced characteristics between the two groups. Propensity scores were estimated using logistic regression by using the following covariates: age, sex, performance status (PS), tumor location, histologic type, tumor stage, tumor differentiation and operation. Statistical analyses were performed using IBM SPSS for Mac, version 23.0. (SPSS, Chicago, IL).

## Results

### Patient characteristics

During the period from January 2015 to May 2017, 212 patients were diagnosed with EC and received radiotherapy. A total of 157 cases (74.1%) were presented at MDT meetings, whereas 55 cases (25.9%) were not presented. There was similar baseline patient and tumor characteristics between the two groups, such as age groups, male/female ratio, PS, tumor stage and location, histological subtype and tumor grade. Significantly more patients in the MDT group received chemotherapy than in the non-MDT group (84.7% vs. 69.1%, *P* = 0.01) (see Table 1).

**Table 1:** Patient, tumor, treatment characteristics before and after propensity score matching

Variables	Before matching				After matching			
	All n = 212	MDT ***n*** = 157	Non-MDT ***n*** = 55	P	All ***n*** = 106	MDT n = 53	Non-MDT ***n*** = 53	P
**Age(y)**	64 (44–89)	64 (44–89)	64 (46–88)	0.52	64 (44–89)	63 (44–89)	65 (46–88)	0.56
**< 60**	66 (31.1%)	44 (28%)	22 (40%)	0.26	38 (35.8%)	18 (34%)	20 (37.7%)	0.33
**60–75**	115 (54.2%)	89 (56.7%)	26 (47.3%)		43 (40.6%)	25 (47.2%)	18 (34%)	
**≥ 75**	31 (14.6%)	24 (15.3%)	7 (12.7%)		25 (23.6%)	10 (18.9%)	15 (28.3%)	
**Sex**								
**Male**	182 (85.8%)	137 (87.3%)	45 (81.8%)	0.32	89 (84%)	45 (84.9%)	44 (83%)	0.79
**Female**	30 (14.2%)	20 (12.7%)	10 (18.2%)		17 (16%)	8 (15.1%)	9 (17%)	
**PS**								
**0–1**	197 (92.9%)	146 (93%)	51 (92.7%)	0.37	98 (92.5%)	49 (92.5%)	49 (92.5%)	1
**2–3**	15 (7.1%)	11 (7%)	4 (7.3%)		8 (7.5%)	4 (7.5%)	4 (7.5%)	
**Location**								
**Neck-Upper**	42 (19.8%)	30 (19.1%)	12 (21.8%)	0.33	21 (19.8%)	10 (18.9%)	11 (20.8%)	0.81
**Middle-Lower**	170 (80.2%)	127 (80.9%)	43 (78.2%)		85 (80.2%)	43 (81.1%)	42 (79.2%)	
**Histologic type**								
**SCC**	196 (92.5%)	145 (92.4%)	51 (92.7%)	0.93	97 (91.5%)	48 (90.6%)	49 (92.5%)	1
**Adeno or others**	16 (7.6%)	12 (7.6%)	4 (7.3%)		9 (8.5%)	5 (9.4%)	4 (7.5%)	
**Stage**								
**I-II**	50 (23.6%)	33 (21.6%)	16 (29.1%)	0.52	30 (28.3%)	14 (26.4%)	16 (30.2%)	0.77
**III**	147 (69.3%)	112 (71.3%)	35 (6360%)		66 (62.3%)	33 (62.3%)	33 (62.3%)	
**IV**	15 (7.1%)	11 (7%)	4 (7.3%)		10 (9.4%)	6 (11.3%)	4 (7.5%)	
**Differentiation**								
**I-II**	67 (31.6%)	54 (34.4%)	13 (23.6%)	0.33	27 (25.5%)	14 (26.4%)	13 (24.5%)	0.91
**III-IV**	57 (26.9%)	41 (26.1%)	16 (29.1%)		28 (26.4%)	13 (24.5%)	15 (28.3%)	
**unknown**	88 (41.5%)	62 (39.5%)	26 (47.3%)		51 (48.1%)	26 (49.1%)	25 (47.2%)	
**Treatment modality**								
**With operation**	104 (49.1%)	77 (49%)	27 (49.1%)	1	50 (47.2%)	24 (45.3%)	26 (49.1%)	0.7
**non-operation**	108 (50.9%)	80 (51%)	28 (50.9%)		56 (52.8%)	29 (54.7%)	27 (50.9%)	
**With chemotherapy**	171 (80.7%)	133 (84.7%)	38 (69.1%)	**0.01**	78 (73.6%)	40 (75.5%)	38 (71.7%)	0.66
**non-chemotherapy**	41 (19.3%)	24 (15.3%)	17 (30.9%)		28 (26.4%)	13 (24.5%)	15 (28.3%)	

*SCC* squamous cell carcinoma, *MDT* multidisciplinary team

### Survival

**Fig. 1:**
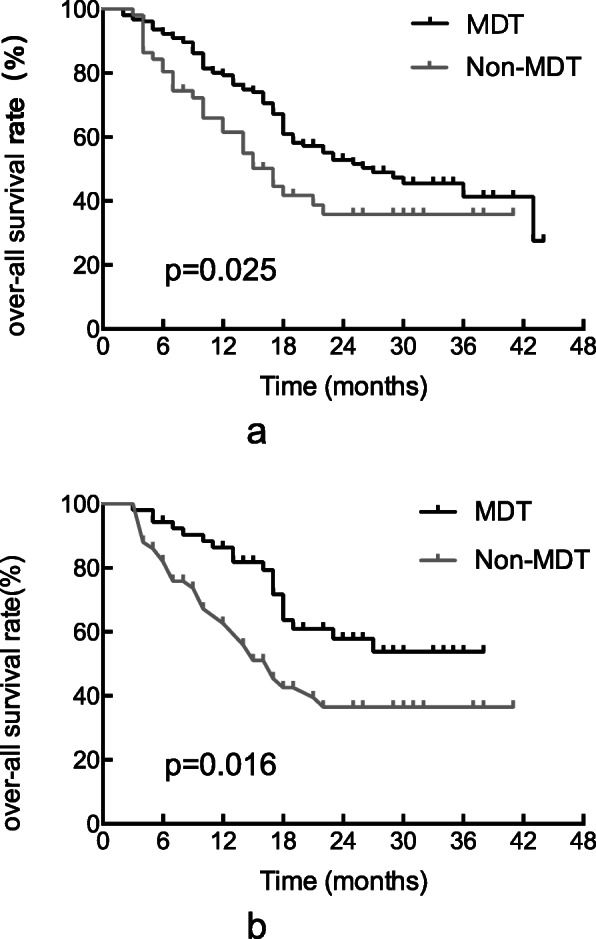
Kaplan–Meier analysis of overall survival of the multidisciplinary team (MDT) and non-MDT groups. a. Before propensity score matching; b. After propensity score matching

The median follow-up time was 26 months. For all patients (*n* = 212), the median OS was 23 months (95% confidence interval [CI], 15.7–30.3 months). The median OS for patients in the MDT group (27 months, 95% CI, 17.5–36.5 months) was significantly longer than that for patients in the non-MDT group (17 months, 95% CI, 12.7–21.3 months, *P* = 0.025). The 1-, 2-, and 3-year survival rates were 79.4, 52.9, and 41.4% in the MDT group and 65.9, 35.8 and 35.8% in the non-MDT group, respectively. The estimated Kaplan-Meier curves for OS are shown in Fig. 1a.

The variables associated with OS in the univariate analysis were PS 2–3 (*P* = 0.001, HR 3.72, 95% CI 2.01–6.89), tumor stage (P = 0.001; for stage III: *P* = 0.002, HR 2.53, 95% CI 1.42–4.51; for stage IV: P = 0.001, HR 4.74, 95% CI 2.09–10.75), surgery (*P* = 0.01, HR 0.59, 95% CI 0.39–0.88) and MDT (*P* = 0.029, HR 0.62, 95% CI 0.340–0.96). In multivariate analysis, MDT (*P* = 0.019, HR 0.59, 95% CI 0.38–0.92), as well as a PS 1–2 and early tumor stage, was associated with an improved OS (see Table 2).

**Table 2:** Risk factors for overall survival using univariate multivariate analysis in patients with EC underwent RT

Variables	univariate			multivariate		
	P	OR	95%CI	P	OR	95%CI
**Age(y)**	0.107					
**< 60**	1					
**60–75**	0.05	0.64	0.41–1.00			
**≥ 75**	0.917	0.97	0.53–1.78			
**Sex**						
**Male**	1					
**Female**	0.056	0.51	0.26–1.02			
**PS**						
**0–1**	1					
**2–3**	0.001	3.72	2.01–6.89	0.002	2.90	1.49–5.62
**Location**						
**Neck-Upper**	1					
**Middle-Lower**	0.646	1.13	0.68–1.88			
**Histologic type**						
**SCC**	1					
**Adeno or others**	0.517	0.76	0.33–1.74			
**Stage**	0.001			0.012		
**I-II**	1					
**III**	0.002	2.53	1.42–4.51	0.004	2.37	1.31–4.29
**IV**	0.001	4.74	2.09–10.75	0.017	3.05	1.22–7.62
**Differentiation**	0.19					
**I-II**	1					
**III-IV**	0.183	1.43	0.85–2.42			
**unknown**	0.077	1.55	0.95–2.51			
**Treatment modality**						
**non-operation**	1					
**With operation**	0.01	0.59	0.39–0.88	0.354	0.81	0.53–1.26
**non-MDT**	1					
**With MDT**	0.029	0.615	0.40–0.95	0.019	0.59	0.38–0.92

*EC* esophageal cancer, *RT* radiotherapy, *SCC* squamous cell carcinoma, *MDT* multidisciplinary team

**Table 3:** Logistic regression model of factors that predicted for receiving chemotherapy

Variables	OR	95% CI	P
**Age(y)**			*P* = 0.176
**< 60**	1.00		
**60–75**	3.57	0.93–13.75	*P* = 0.064
**≥ 75**	2.27	0.75–6.83	*P* = 0.147
**Sex**			
**Male**	1.00		
**Female**	0.32	0.12–0.83	***P*** **= 0.020**
**PS**			
**0–1**	1.00		
**2–3**	0.14	0.04–0.52	**P = 0.006**
**Location**			
**Neck-Upper**	1.00		
**Middle-Lower**	0.72	0.25–2.11	*P* = 0.546
**Histologic type**			
**SCC**	1.00		
**Adeno or others**	0.50	0.12–2.03	*P* = 0.334
**Stage**			*P* = 0.113
**I-II**	1.00		
**III**	2.75	1.06–7.11	***P*** **= 0.037**
**IV**	2.22	0.36–13.83	*P* = 0.394
**Differentiation**			*P* = 0.088
**I-II**	1.00		
**III-IV**	3.86	1.16–12.84	***P*** **= 0.028**
**unknown**	1.73	0.47–6.37	*P* = 0.408
**Treatment modality**			
**non-operation**	1.00		
**With operation**	1.29	0.33–5.07	*P* = 0.719
**non-MDT**	1.00		
**With MDT**	3.03	1.29–7.08	***P*** **= 0.011**

*SCC* squamous cell carcinoma, *MDT* multidisciplinary team

### Analysis of chemotherapy treatment

MDT patients received more chemotherapy than non-MDT patients, especially patients aged 60–75 years old (86.5 and 65.4%, respectively, *P* = 0.02), with SCC (85.5 and 70.6%, respectively, P = 0.02), with middle-low thoracic tumors (85 and 67.4%, respectively, *P* = 0.01), with stage III tumors (90.2 and 65.7%, respectively, P = 0.01), with unknown differentiation (82.3 and 61.5%, respectively, *P* = 0.04), and who did not undergo surgery (82.5 and 64.3%, respectively, *P* = 0.05). We used logistic regression analysis to predict the risk factors for receiving chemotherapy. MDT (P = 0.01, OR 3.03, 95% CI 1.29–7.08), as well as the male sex (*P* = 0.02, OR 3.16), a low PS (*P* = 0.006, OR 6.99), stage III tumors (P = 0.04, OR 2.75), and differentiation III-IV (*P* = 0.03, OR 3.86), was an independent predictor of receiving chemotherapy (see Table 3). There was no overall survival benefit in the chemotherapy group (23 months, 95% CI, 16.2–29.8 months) compared to no chemotherapy group.(22 months, 95% CI, 6.17–37.8 months, *P* = 0.43).

### PSM

To identify the impact of MDT on OS without differences in the treatment options, PSM was performed to keep all baseline characteristics in balance. Considering that whether a patient receives chemotherapy depends on MDT or individual radiation oncologist recommendation. We believe that chemotherapy may be not the condition at baseline. After matching, there was still significant difference in OS between the two groups (median OS was not reached in the MDT group and 17 months in the non-MDT group (*P* = 0.016)) (see Fig. 1b).

## Discussion

The modern management strategies for patients with esophageal cancer requires a multidisciplinary approach that involves surgeons, medical oncologists, gastroenterologists, radiologists, radiation oncologists and pathologists [7–10]. An MDT improves the staging accuracy, treatment selection [4] and outcomes after surgery for patients with EC [3]. The issue of whether to form an MDT for EC patients who underwent radiotherapy has not yet been discussed. Here, we found that with similar patient characteristics and tumor conditions, MDT was associated with a significantly better OS than non-MDT for EC patients undergoing RT.

Several studies have assessed the effect of MDT on patients diagnosed with EC. Davies et al. suggested that the clinical stage determined by an MDT was more accurate than the individual clinical stage determined with T and N staging, which in turn led to more appropriate treatment selections for EC patients. The MDT recommendations differed from the initial treatment plan in 20–26% EC patients [11, 12], which means that there are some differences in treatment option choices between the with MDT and non-MDT groups.

Combined chemotherapy increases the survival of patients with EC receiving definitive treatment (chemoradiotherapy) compared with RT alone [13]. In the context of applying adjuvant radiotherapy in China, chemotherapy is also commonly used in patients with positive nodes [14, 15]. However, some patients do not receive chemotherapy because of a poor PS, comorbidities, unwillingness or the recommendation from doctors.

We found that significantly more patients with MDT received chemotherapy combined with radiotherapy than patients without MDT, especially patients with the following characteristics: 60–75 years old, middle-low thoracic tumor, stage III tumor, and did not undergo surgery. By showing that these patients need chemotherapy based on the tumor characteristics, an MDT can enable oncologists to confidently administer chemotherapy and allow patients to be confident in receiving combination therapy. This is supported by van Hagen and his colleagues who found that 98% of EC patients adhered to the multidisciplinary tumor board meeting recommendations [16]. After balancing the characteristics at baseline in two groups by PSM, the overall survival advantage in MDT group were more obvious. Additionally, we find that MDT is an independent predictor for receiving chemotherapy by using logistic regression analysis. Take together, there is a significantly improved survival for MDT managed patients when controlling the baseline characteristics, and the survival benefits might be due to the use of multimodality therapy after MDT.

The main limitation of our study is that the patients were collected in a retrospective manner from a single institution, so the outcomes inherently have biases. Nevertheless, these results gave us the opportunity to show whether and how MDT affected the survival of patients who underwent radiotherapy. We found that MDT can reasonably use treatment methods to fully maximize the benefits for patients.

## Conclusion

MDT can improve the overall survival for patients with esophageal cancer receiving radiotherapy. This suggest that treatment decisions for such patients should be discussed within a multidisciplinary team.

## Data Availability

Not applicable.

## Abbreviations

RT: Radiotherapy
MDT: multidisciplinary team
EC: esophageal cancer
PSM: Propensity score matching
OS: overall survival rate
SCC: squamous cell carcinoma
ORs: odds ratios
PS: performance status
